# Solar system exploration via comparative planetology

**DOI:** 10.1038/s41467-020-18126-z

**Published:** 2020-08-27

**Authors:** Karl-Heinz Glassmeier

**Affiliations:** Institut für Geophysik und extraterrestrische Physik, Technische Universität Braunschweig, Mendelssohnstrasse 3, 38106 Braunschweig, Germany

**Keywords:** Astronomy and astrophysics, Space physics

## Abstract

Knowing about the diversity of planetary processes is of paramount importance for understanding our planet Earth. An integrated, comparative planetology approach is required to combine space missions, autonomous surface exploration, sample return laboratories, and after-mission data exploitation.

## Why should we explore the solar system?

Because we can. In the past 60 years we have witnessed a most remarkable adventure: the in-situ exploration of our solar system. Space missions like the Voyagers^1^, Magellan^2^, Giotto^3^, Cassini–Huygens^4^, or, more recently, Rosetta^5^, New Horizon^6^, Hayabusa (2)^7^, Parker Solar Probe^8^, OSIRIS-Rex^9^, and now Solar Orbiter^10^ allow(ed) us to visit the Sun, all planets and many of their moons as well as comets, asteroids, and even Kuiper belt objects. There is no reason to stop this story of success, to which also the Hubble Space Telescope added immensely.

However, did we learn for our human society? Yes, and the key term here is comparative planetology, or, more appropriately, comparative Earth science. Often, comparisons of more and different objects are necessary to reach a comprehensive understanding. This holds for processes in and on Earth as well. Living on Earth reached a nonlinear stage. We are no more just using terrestrial resources. We are changing the resource bearing environment in a dramatic way, reaching tipping points and crossing planetary boundaries^11^. An important source of information on this negative feedback is global observations using satellites and space science. Earth observation with missions like ENVISAT deliver necessary contingent information and process understanding on global change, either natural or human made.

**Fig. 1:**
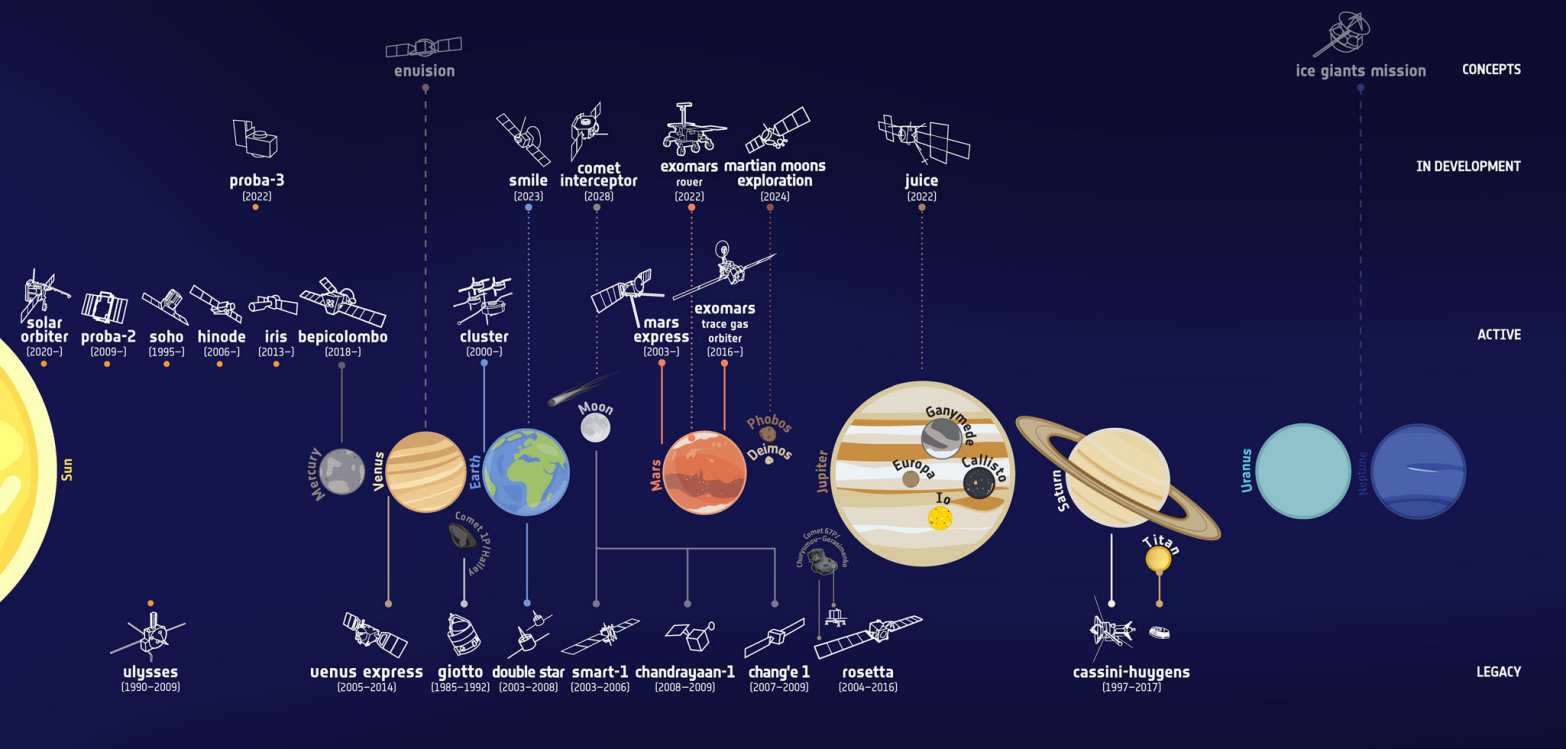
Numerous past, present, and future space missions are the backbone and mining area of comparative planetology. Not only large organisations such as NASA and ESA, whose activities are displayed here, are contributing. The recent launches of three missions to Mars indicate the global interest in planetology (Credit: ESA).

But, comparative Earth science is mandatory for deeper insight. The diversity of geological processes in the solar system is fascinating. Earth currently operates an ecosystem which supports biological activity. Since the age of space exploration, we know that Earth is the only planetary body in our solar system with human life forms. The slowly rotating planet Mercury is too hot on its dayside to support life. Venus has a very thick atmosphere with climate conditions hostile to biogenic activity. Saturn’s satellite Titan is covered by a thick nitrogen atmosphere with temperature about −180 °C, methane clouds and rain, a very hostile environment as well. Did other planetary bodies reach their natural planetary boundaries? Studying the strange worlds in our solar system puts terrestrial geological processes into a proper perspective. The various planetary objects studied in the past, currently under investigation, and object of future space explorer missions, are not only entangled by the gravitational field of the Sun, but also by the need for comparative planetology (Fig. 1). The term habitable zone was coined to describe regions around stars where carbon-based life is possible, based on our new, space-based knowledge. Doing space exploration is an important means for human and scientific self-assertion.

## Ice giant exploration required

There are a few stepping stones into our space future. First, there is missing knowledge on two major objects, Uranus and Neptune. Only Voyager 2 did flybys. Extreme worlds were detected. Both possess an internally generated magnetic field of, probably, dynamo origin. Their field topologies are much more complex than that one of Earth. Surface features indicate a variety of geologic processes we do not have a decent understanding of. It is a must to send out orbiters around Uranus and Neptune to get a better knowledge on our ice giants.

## Robotic surface exploration

There are no alternatives to in-situ measurements at the surfaces of planetary objects. We achieved this for the Moon, Mars, comet 67 P/Churyumov-Gerasimenko, and asteroids like (433) Eros, (162173) Ryugu, and soon (101955) Bennu. We definitely need to proceed along these lines. Surface truth is inevitable in deepening our understanding. The recent observations of InSight of only moderate seismic activity on Mars^12^ pose questions on the differences of structure and dynamics of the terrestrial planets. Without surface measurements we would not have had any chance to tackle this question, to the benefit of understanding the diversity of planetary processes.

Robotic surface exploration, my second stepping stone, is the only realistic tool in planetary science for in-situ surface observations of the more exotic worlds. And it is quite a demanding enterprise. New technologies need to be developed and tested. The Moon is to be used as a most interesting future testbed. It is reachable by humans within days as the first lunar landing, whose 50th anniversary we just celebrated, demonstrated. Our close cosmic companion provides harsh enough environments, allowing human interference with autonomous systems to be developed and tested. Fleets of self-aware robotic explorers need to be designed for the exploration of the other planetary body surfaces. Light travel time limits an efficient communication with any robotic device in the outer solar system. The lunar village is the future international space and technology development centre for such self-aware robotic explorers.

## More virtual reality use

**Fig. 2:**
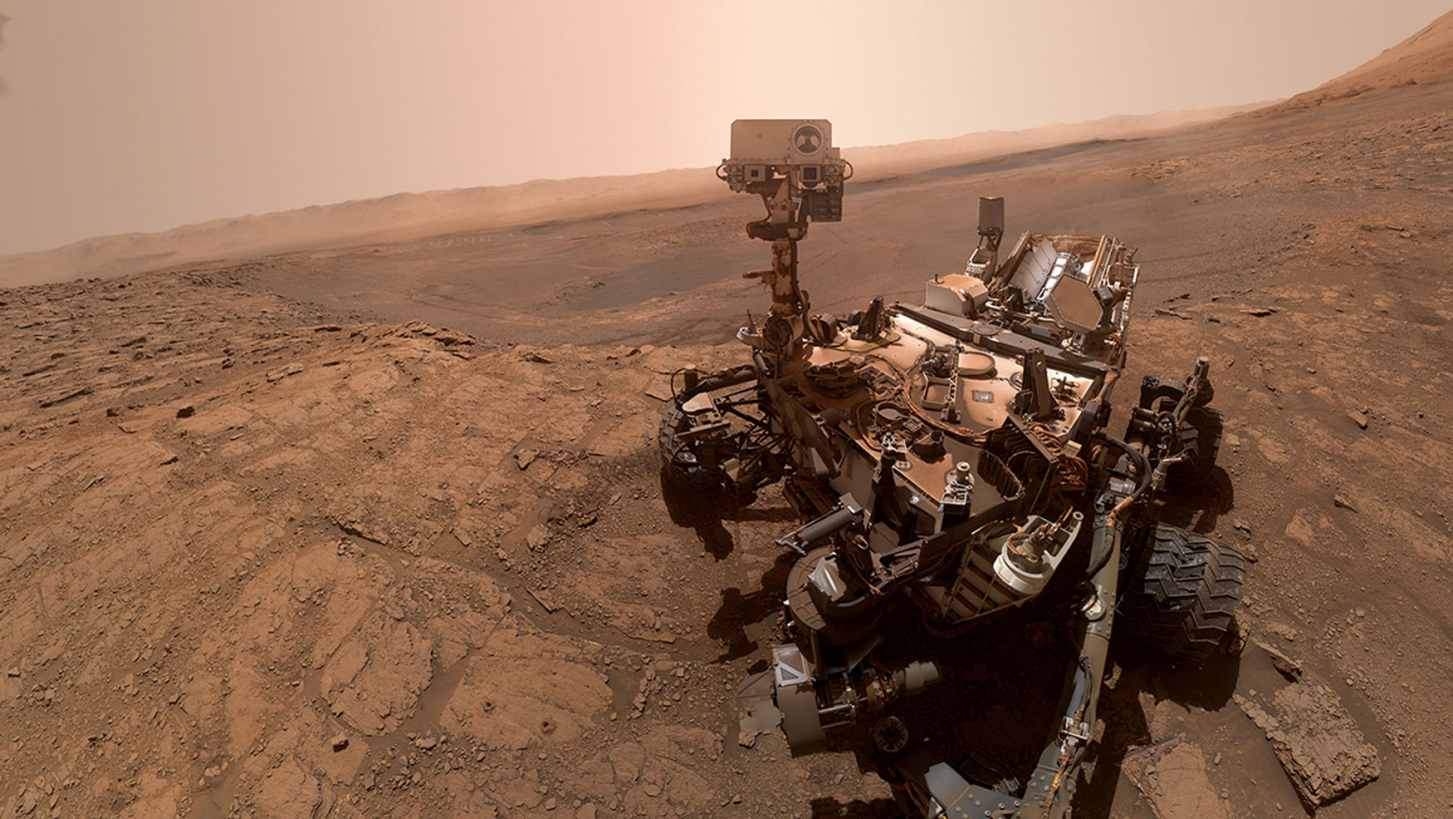
Selfie of NASA’s Curiosity rover, taken on Oct. 11, 2019. The geologic structure in the background is the northern rim of the Gale Crater. More advanced, self-aware rovers are necessary to provide future geologic information from the hostile surfaces of planetary bodies (Credit: NASA).

The recent successful launch of a private company’s manned-spacecraft to the International Space Station may trigger dreams for unlimited travel to endless frontiers. Humans may be able to travel to Mars. But, is there any chance for a human to reach the surface of Enceladus, this mysterious Kronian moon? In science fiction yes, in reality not. Of course, one would like to witness a cold geyser eruption on this moon’s surface! And we should not give up this dream! If you touch a stone the sensation is mediated via tactile sensors and our nervous system to our brain. In a similar way we should use autonomous systems to sense planetary surfaces and send back vast amounts of information to our home planet, pictures, acoustic information, chemical analyses, and even tactile information^13^. Combine all this information in a virtual reality lab for further in-depth processing, human exploration, and curiosity driven adventure (Fig. 2). My third stepping stone: let us intensive the use of virtual reality tools like in other training environments, providing experience on demand^14^.

## More sample return missions required

There may be analyses too complex to be done in a robotic laboratory. A fourth stepping stone is sample return missions. Material probes need to brought back from the planetary surfaces for in-depth analysis on Earth. First, this was done successfully by Apollo 11 in 1969. Currently, hope rests on Hayabusa (2)^7^ and OSIRIS-Rex^9^ and their returned samples. Sample return missions require the build-up of appropriate planetary sample analysis laboratories^15^. This is an international, community wide activity, strongly to be supported as an integral part of any planetary surface landing mission. We jointly need to go to the surfaces and bring back information and samples.

## Internationally coordinated funding for science exploitation

Besides technological and scientific demands structural problems need to be attacked in the upcoming space years, defining my fifth stepping stone towards an integrated solar system research enterprise. There is an urgent need to provide more financial resources for after-mission science data exploitation, at least in the European sector. NASA, for example, foresees such funding possibilities. However, the European Space Agency, according to its charter, merely supports hardware developments, launch possibilities, spacecraft operations, and data archiving. Scientific exploitation of data collected is entirely up to European national science funding agencies. This very often leads to unhealthy situations. For instance, Rosetta, Europe’s planetary flagship, finished operations in autumn 2016. After a data archiving phase, the mission was coming to an end in September 2017. However, much of the observations have not yet been analysed. The scientific mission is actually not finished. But very limited European wide coordinated effort has been initiated to secure the further exploitation of the data, due to a missing science exploitation plan. No private company would dare to invest 1.5 billion euros, and cutting the return-of-investment line that early. At least 2 percent of the overall mission investment should be reserved to coordinate the after mission operation science exploitation and analysis! Such funds should also be used to foster citizen science projects.

## Knowledge transfer

My sixth stepping stone covers a further important aspect: space technology and science differ from other scientific fields. Space projects are long lasting projects. The Voyagers were launched in 1977, were still operational in 2012 and 2018, when they left the solar system. The Rosetta mission planning and completion took more than 25 years. Knowledge transfer becomes an issue here. The return of investment for such lifelong technical/scientific projects needs a guarantee. Knowledge transfer requires careful consideration. While space agencies usually are legally well founded in their countries the situation at university and research institutes is different. At universities, for example, faculties decide in an autonomous way on the nomination of successorships. This does not imply that any long-term mission is supported in any case. As a consequence, each future space project needs to be accompanied by an overarching mission completion plan, long-term contracts between the various partners. The need for new insight must be paired with foresight.

These six stepping stones should be a basis for an integrated decadal space exploration enterprise. Space exploration, it is for understanding our planet Earth. Reaching out for the solar system is not only because we can.
